# A mobile-based pregaming drinking prevention intervention for college students: study protocol for a randomized controlled trial

**DOI:** 10.1186/s13722-022-00314-5

**Published:** 2022-06-18

**Authors:** Eric R. Pedersen, Justin F. Hummer, Jordan P. Davis, Reagan E. Fitzke, Nina C. Christie, Katie Witkiewitz, John D. Clapp

**Affiliations:** 1 Keck School of Medicine, Department of Psychiatry and Behavioral Sciences, USC Institute for Addiction Science, University of Southern California, 2250 Alcazar Street, Suite 2200, Los Angeles, CA 90033 United States; 2 RAND Corporation, Santa Monica, United States; 3 Suzanne Dworak-Peck School of Social Work; USC Center for Artificial Intelligence in Society; USC Center for Mindfulness Science; USC Institute for Addiction Science, University of Southern California, Los Angeles, United States; 4 Department of Psychology, University of Southern California, Los Angeles, United States; 5 Department of Psychology, University of New Mexico, Albuquerque, United States; 6 Suzanne Dworkak-Peck School of Social Work; Department of Population and Public Health Sciences, Keck School of Medicine; USC Institute for Addiction Science, University of Southern California, Los Angeles, United States

**Keywords:** Alcohol, Intervention, Normative feedback, Young adults, Prepartying, Predrinking

## Abstract

**Background:**

Pregaming is a high-drink context popular among college students that often leads to elevated blood alcohol levels and negative consequences. Over 15 years of research studies have demonstrated that pregaming represents one of the riskiest known behaviors among college students, yet no pregaming-specific interventions have been developed to help prevent this behavior. General brief interventions for students do not reduce pregaming behavior and may not be appropriate, as they do not help students develop skills unique to the pregaming context that could help them drink less. We developed a brief, mobile-based intervention that is proposed to prevent heavy drinking during pregaming for college students, with the ultimate goal that behavioral reductions in this risky practice will ultimately affect global drinking and prevent consequences.

**Methods/Design:**

The intervention, Pregaming Awareness in College Environments (PACE), was developed by combining two innovations to facilitate behavior change: (1) a mobile-based application that increases accessibility, is easy and engaging to use, and broadens the reach of the intervention content and (2) personalized pregaming-specific intervention content with harm reduction and cognitive behavioral skills proven to be mechanisms preventing and reducing heavy drinking among college students. After a develop and beta-test phase, we propose to test the efficacy of PACE in a preliminary randomized controlled trial with 500 college students who pregame at least once per week. Pregaming, general drinking, and alcohol-related consequences outcomes will be examined in the immediate (2 weeks post-intervention) and short-terms (six and 14-week post-intervention). We will also evaluate moderator effects for age, sex, and heaviness of drinking to allow for more refined information for a planned larger test of the intervention to follow this initial trial of PACE.

**Discussion:**

This pregaming intervention clinical trial, if found to be efficacious, will culminate with an easily-disseminated mobile-based intervention for college student drinkers. It has the potential to reach millions of college students, perhaps as a clinical tool used by college counseling centers as an adjunct to formal care or as a preventive tool for first-year students or other high-risk groups on campus.

*Trial registration*: ClinicalTrials.gov Identifier NCT04016766.

## Background

Despite substantial intervention efforts to reduce college student drinking and resulting consequences [1–3], both continue to be national public health concerns and thus, important foci of prevention and intervention efforts [4–6]. National data from 2019 indicate most college students drink alcohol (78% annual prevalence, 62% past month prevalence), 32% engage in heavy episodic drinking (five or more drinks in a row in the past two weeks), and 35% report being drunk in the past month [7]. These national data also indicate that “high intensity drinking” is common, with 12% of college students reporting consumption of 10 or more drinks in a row on an occasion during the past two weeks [8, 9]. Prevalence rates of high intensity drinking increase from age 18 to 22, with the steepest increases occurring over time for college students compared to young adults not attending college [8]. The consequences of heavy college drinking are well documented and include academic problems, physical injuries and fights, risky sexual behavior and sexual assaults, memory blackouts and passing out, sustained cognitive deficits, alcohol poisoning, and even death [10].

Several interventions have been designed to address heavy drinking and related consequences among college students. These interventions are often brief in order to be acceptable by students and feasible to deliver to large numbers of students in universal or selective prevention efforts. Many college students do not consider their drinking to be problematic or something that needs to be changed [9]; many are in a stage of change where they are not yet considering making any changes to their drinking (called the *precontemplation* stage in the transtheoretical model) or are considering making some changes but have no plan of action to make such changes (called *contemplation*) [11]. Most of the interventions use components first documented together in the Brief Alcohol Screening and Intervention for College Students (BASICS) approach [12], typically combine strategies from Motivational Interviewing (MI), [13] (e.g., strategies to increase motivation to change/reduce risky behavior), cognitive behavioral skills training [14] (e.g., drink refusal skills), harm reduction strategies [15, 16] (e.g., protective strategies for limiting consumption), and personalized feedback (e.g., review of personalized consequences such as alcohol calories consumed each week). Yet, studies on in-person BASICS and adaptations of the program’s components into group, computer, web-based, and mobile formats have demonstrated modest effects at best [3, 17–19], which has sparked debate about whether such brief interventions are clinically meaningful or impactful on a broader public health scale [20–23]. It is becoming increasingly clear that brief interventions for college students need to be refined or enhanced in order to have a larger impact on changing the ingrained heavy drinking culture present on many college campuses today.

Although brief interventions with students often include aspects based on a relapse prevention approach, wherein they identify high-risk situations and apply specific skills to manage these situations with minimal or no use of alcohol [12, 14, 16], global interventions can be vague with regard to when to use certain skills. The college context is diverse, with individual student’s drinking levels varying between specific contexts (e.g., drinking more at a bar versus at a party, drinking less on a Wednesday than on a Thursday) [24, 25]. To expand upon brief interventions that target behavior at a global level, contemporary prevention programs prepare students for the inevitable risks associated with specific high-risk drinking events. Such events include spring break, 21st birthdays, holidays such as St. Patrick’s Day, and study abroad trips [26–31], which are periods where students drink at heavy or high-intensity levels, placing them at even greater risk than during a typical week on campus. Thus, these “event-specific” approaches lay out a clear framework for the specific skills that students can implement in discrete circumstances, often in preparation for an upcoming risky event where heavy drinking is likely to occur.

Event-specific prevention programs have been tested with promising effects, [32–34] and they represent an approach to combat college drinking beyond a global level, wherein students learn specific skills to prepare for an event anticipated to involve risky drinking. Targeted preventive education reduces ambiguity about how, when, and where to use a learned skill, which can thereby increase the successful implementation of that skill in real life. Although event-specific prevention outcomes are generally positive, their effects are often short-lived (e.g., spring break is just week, 21st birthdays are just one day). Less clear is if modifying drinking behavior in one specific context (e.g., a 21st birthday celebration) can translate to sustained behavior change in other diverse drinking contexts. Ideally, an event-specific prevention program would target a high-risk drinking behavior involved in most drinking contexts so that event-specific skills learned in the program could be employed more frequently and broadly.

One such frequent, yet risky, drinking behavior that has received growing empirical attention and heightened concern is called “pregaming”. Pregaming’s etymology stems from its roots in “tailgating” prior to sporting events, but local and regional vernacular has evolved to include terms such as prepartying, preloading, predrinking, and front-loading. The behavior has expanded well beyond tailgating-specific events, as students report pregaming across a number of different drinking contexts, such as before going to bars, parties, concerts, football games, or on dates; with friends or alone; while playing drinking games; while getting ready to go out; and even while driving to their destination for the night [35–38]. Pregaming is prevalent among American college students, ubiquitous across college drinking contexts, and consistently involves or leads to high intensity drinking [35, 39–43]. During pregaming, people consume multiple drinks during a brief period prior to going to an event or social gathering where more alcohol is typically consumed. It is highly prevalent among students, with over 40% of all college students reporting past month pregaming and past month prevalence rates among student drinkers ranging from 50 to 85% across studies [39, 44]. Pregaming is not specific to U.S. college students, as the behavior has been studied among young people in several other countries, such as Switzerland, the United Kingdom, and Australia, with similar findings related to its prevalence and risks [38, 45, 46].

Among U.S. students, around one-third or more of all drinking days involve pregaming [35, 40, 47, 48], with students typically consuming between three to five drinks within just one to two hours [35, 49]. Such quick-paced drinking can lead to high blood alcohol levels (BALs), which are reached even before students leave for their intended destination, at which point they often go on to drink more. To wit, most pregaming events involve further drinking once students reach their intended destination [49, 50], which typically results in a total drink count for the night indicative of high intensity drinking (i.e., 10 or more drinks) [51]. High BALs can lead to negative consequences on a night out, and severe alcohol-related consequences have been linked to pregaming drinking, including hospitalizations, regretted sex, driving after drinking, blacking out, and passing out [35, 40, 43, 49, 52–55]. Further, students drink more on pregaming nights than on non-pregaming nights [35, 40, 43, 49, 54, 55], and longitudinal research shows that pregaming frequency predicts heavy drinking behavior and alcohol-related consequences even up to one year later [42], suggesting long term impacts on risky alcohol use trajectories.

Given the risks associated with pregaming, it would be important for interventions that target drinking globally to also affect changes in pregaming behavior specifically. Yet, interventions that target general drinking patterns do not show effects on pregaming behavior. For example, one published study evaluated pregaming outcomes after a global, brief, group intervention with mandated students and failed to find significant reductions in pregaming post-intervention, even if pregaming was mentioned (albeit infrequently) by students in the discussion portions of the intervention [56]. Another study found that a general alcohol-reduction intervention for student-athletes did not reduce athletes’ pregaming behavior one- and four-months post-intervention [57]. Thus, approaches specifically targeting pregaming may be necessary for reductions to occur in pregaming-specific heavy drinking. Such an approach can selectively target the specific behavior known to lead to subsequent consequences and heavy drinking both on the pregaming day and more generally.

Only three studies to our knowledge have examined the effects of a pregaming-specific intervention on pregaming behaviors. There is promise from a small experimental study that found that providing female students with fabricated normative information that other students pregame less frequently than they perceived prevented pregaming during a subsequent drinking occasion [58]. A second study by Caudwell and colleagues [59] examined the efficacy of two online interventions that shared Australian national drinking guidelines with students who were assigned to either complete an exercise based on autonomy support (e.g., reminders that drinking less during pregaming could help reduce negative consequences) or on implementation intentions (e.g., intentions to use protective behavioral strategies to limit consumption, like drinking a glass of water after consuming a pregaming drink). Participants in all conditions (including an intervention condition where participants completed both exercises and a control group that received neither intervention) did not differ at a four-week follow-up in their reductions in pregaming drinking and alcohol-related consequences. A third study by Cadigan and colleagues [60] evaluated a very brief text-message intervention delivered to students prior to attending a tailgating event at a college football game, finding that students who received the intervention consumed fewer drinks and reached lower estimated BALs than those in a control condition. Moreover, the intervention, though targeted toward a specific one-time football game event, was associated with fewer alcohol-related problems one month later. The findings lend support to the notion that helping students change how they drink during one specific high-risk event may translate to lower risk drinking during other events in the near term.

## The present study

The prior studies evaluating pregaming interventions are promising, but perhaps limited due to their brevity and focus on targeting perceived norms or providing prompts/reminders only, thus not incorporating multiple components of brief alcohol interventions known to help students reduce heavy drinking [18]. Without multiple evidence-based components that have been tested in interventions targeting broader, global drinking behaviors, lasting change may be difficult to obtain. Changing the way students drink during pregaming could not only prevent heavy drinking and its consequences following the pregaming event on a particular night, but it could subsequently reduce overall drinking behavior and alcohol-related consequences more globally for an individual. We designed a brief mobile intervention to address the high-risk drinking behavior of pregaming, targeting the multitude of different pregaming contexts (e.g., before going to a concert, party, bar, or date) beyond tailgating before football games. Targeting a high-frequency event that occurs in many different contexts has potential for greater impact on total consumption than other event-specific interventions (e.g., those targeted on 21st birthdays or spring breaks). As such, an empirically-supported approach focusing on pregaming, a behavior known to lead to both event-specific and global consequences, would improve upon existing global and event-specific interventions. Table 1 contains Standard Protocol Items: Recommendations for Interventional Trials (SPIRIT) recommended sections within this protocol.

**Table 1:** SPIRIT 2013 Checklist: Recommended items to address in a clinical trial protocol and related documents

Section/item	Item No	Description	Addressed on page number
Administrative information			
Title	1	Descriptive title identifying the study design, population, interventions, and, if applicable, trial acronym	1
Trial registration	2a	Trial identifier and registry name. If not yet registered, name of intended registry	3
	2b	All items from the World Health Organization Trial Registration Data Set	2–3
Protocol version	3	Date and version identifier	1
Funding	4	Sources and types of financial, material, and other support	23
Roles and responsibilities	5a	Names, affiliations, and roles of protocol contributors	1
	5b	Name and contact information for the trial sponsor	23
	5c	Role of study sponsor and funders, if any, in study design; collection, management, analysis, and interpretation of data; writing of the report; and the decision to submit the report for publication, including whether they will have ultimate authority over any of these activities	23
	5d	Composition, roles, and responsibilities of the coordinating centre, steering committee, endpoint adjudication committee, data management team, and other individuals or groups overseeing the trial, if applicable (see Item 21a for data monitoring committee)	23
Introduction			
Background and rationale	6a	Description of research question and justification for undertaking the trial, including summary of relevant studies (published and unpublished) examining benefits and harms for each intervention	4–9
	6b	Explanation for choice of comparators	14–15
Objectives	7	Specific objectives or hypotheses	8–9
Trial design	8	Description of trial design including type of trial (eg, parallel group, crossover, factorial, single group), allocation ratio, and framework (eg, superiority, equivalence, noninferiority, exploratory)	
			9–10
Methods: participants, interventions, and outcomes			
Study setting	9	Description of study settings (eg, community clinic, academic hospital) and list of countries where data will be collected. Reference to where list of study sites can be obtained	8–10
Eligibility criteria	10	Inclusion and exclusion criteria for participants. If applicable, eligibility criteria for study centres and individuals who will perform the interventions (eg, surgeons, psychotherapists)	10
Interventions	11a	Interventions for each group with sufficient detail to allow replication, including how and when they will be administered	10–15
	11b	Criteria for discontinuing or modifying allocated interventions for a given trial participant (eg, drug dose change in response to harms, participant request, or improving/worsening disease)	Not available
	11c	Strategies to improve adherence to intervention protocols, and any procedures for monitoring adherence (eg, drug tablet return, laboratory tests)	Not available
	11d	Relevant concomitant care and interventions that are permitted or prohibited during the trial	10
Outcomes	12	Primary, secondary, and other outcomes, including the specific measurement variable (eg, systolic blood pressure), analysis metric (eg, change from baseline, final value, time to event), method of aggregation (eg, median, proportion), and time point for each outcome. Explanation of the clinical relevance of chosen efficacy and harm outcomes is strongly recommended	
			16–18
Participant timeline	13	Time schedule of enrolment, interventions (including any run-ins and washouts), assessments, and visits for participants. A schematic diagram is highly recommended (see Figure)	25
Sample size	14	Estimated number of participants needed to achieve study objectives and how it was determined, including clinical and statistical assumptions supporting any sample size calculations	9
Recruitment	15	Strategies for achieving adequate participant enrolment to reach target sample size	9–10
Methods: Assignment of interventions (for controlled trials)			
Allocation:			
Sequence generation	16a	Method of generating the allocation sequence (eg, computer-generated random numbers), and list of any factors for stratification. To reduce predictability of a random sequence, details of any planned restriction (eg, blocking) should be provided in a separate document that is unavailable to those who enrol participants or assign interventions	10
Allocation concealment mechanism	16b	Mechanism of implementing the allocation sequence (eg, central telephone; sequentially numbered, opaque, sealed envelopes), describing any steps to conceal the sequence until interventions are assigned	Not available
Implementation	16c	Who will generate the allocation sequence, who will enrol participants, and who will assign participants to interventions	Not available
Blinding (masking)	17a	Who will be blinded after assignment to interventions (eg, trial participants, care providers, outcome assessors, data analysts), and how	Not available
	17b	If blinded, circumstances under which unblinding is permissible, and procedure for revealing a participant’s allocated intervention during the trial	Not available
Methods: data collection, management, and analysis			
Data collection methods	18a	Plans for assessment and collection of outcome, baseline, and other trial data, including any related processes to promote data quality (eg, duplicate measurements, training of assessors) and a description of study instruments (eg, questionnaires, laboratory tests) along with their reliability and validity, if known. Reference to where data collection forms can be found, if not in the protocol	12–14
	18b	Plans to promote participant retention and complete follow-up, including list of any outcome data to be collected for participants who discontinue or deviate from intervention protocols	10
Data management	19	Plans for data entry, coding, security, and storage, including any related processes to promote data quality (eg, double data entry; range checks for data values). Reference to where details of data management procedures can be found, if not in the protocol	10–11
Statistical methods	20a	Statistical methods for analysing primary and secondary outcomes. Reference to where other details of the statistical analysis plan can be found, if not in the protocol	15–16
	20b	Methods for any additional analyses (eg, subgroup and adjusted analyses)	15–164
	20c	Definition of analysis population relating to protocol non-adherence (eg, as randomised analysis), and any statistical methods to handle missing data (eg, multiple imputation)	
			15–16
Methods: Monitoring			
Data monitoring	21a	Composition of data monitoring committee (DMC); summary of its role and reporting structure; statement of whether it is independent from the sponsor and competing interests; and reference to where further details about its charter can be found, if not in the protocol. Alternatively, an explanation of why a DMC is not needed	Not available
	21b	Description of any interim analyses and stopping guidelines, including who will have access to these interim results and make the final decision to terminate the trial	Not available
Harms	22	Plans for collecting, assessing, reporting, and managing solicited and spontaneously reported adverse events and other unintended effects of trial interventions or trial conduct	Not available
Auditing	23	Frequency and procedures for auditing trial conduct, if any, and whether the process will be independent from investigators and the sponsor	Not available
Ethics and dissemination			
Research ethics approval	24	Plans for seeking research ethics committee/institutional review board (REC/IRB) approval	10
Protocol amendments	25	Plans for communicating important protocol modifications (eg, changes to eligibility criteria, outcomes, analyses) to relevant parties (eg, investigators, REC/IRBs, trial participants, trial registries, journals, regulators)	Not available
Consent or assent	26a	Who will obtain informed consent or assent from potential trial participants or authorised surrogates, and how (see Item 32)	10
	26b	Additional consent provisions for collection and use of participant data and biological specimens in ancillary studies, if applicable	Not available
Confidentiality	27	How personal information about potential and enrolled participants will be collected, shared, and maintained in order to protect confidentiality before, during, and after the trial	9–10
Declaration of interests	28	Financial and other competing interests for principal investigators for the overall trial and each study site	23
Access to data	29	Statement of who will have access to the final trial dataset, and disclosure of contractual agreements that limit such access for investigators	23
Ancillary and post-trial care	30	Provisions, if any, for ancillary and post-trial care, and for compensation to those who suffer harm from trial participation	Not available
Dissemination policy	31a	Plans for investigators and sponsor to communicate trial results to participants, healthcare professionals, the public, and other relevant groups (eg, via publication, reporting in results databases, or other data sharing arrangements), including any publication restrictions	Not available
	31b	Authorship eligibility guidelines and any intended use of professional writers	Not available
	31c	Plans, if any, for granting public access to the full protocol, participant-level dataset, and statistical code	23

## Methods/Design

### Procedures

For this study, we propose to develop and test a brief mobile (i.e., mobile phone-friendly website) intervention directly targeting pregaming among college students. First, we developed the intervention content and programmed the online intervention. We then beta-tested the developed program with heavy drinking college students who reported pregaming at least once per week on average and gathered feedback regarding feasibility and acceptability of intervention content. Final edits to the brief intervention were made based on these students’ feedback. Next, we will conduct a randomized controlled trial (RCT) of the intervention with approximately 500 college students from one private southern California university, assigning half to intervention and half to control. We will evaluate immediate term (from two weeks pre-intervention to two weeks post-intervention) and short term (from one month pre-intervention to six weeks and 14 weeks post-intervention) drinking during pregaming, overall drinking, and consequences.

**Fig. 1:**
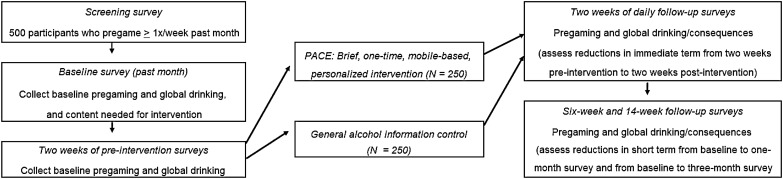
Flow of the RCT

For the RCT, we will recruit (1) full time undergraduate students at the university who are (2) between the ages of 18 and 24 and (3) report pregaming at least once per week in the past month. No other eligibility criteria beyond these will use in an effort to obtain students who pregame but who may not be considering making changes to their drinking. Participants will be recruited by emails sent to a random selection of undergraduates, via a list of student emails obtained from the university’s registrar. Participants will complete a screening questionnaire to determine if they meet eligibility criteria. Those screening into the study will complete a 20-min baseline survey, followed by two weeks of daily surveys. Participants will then be randomized to receive the brief pregaming intervention or a control condition program (randomized by computer-generated random numbers). After viewing one or the other program, they will complete two additional weeks of daily surveys, followed by a 20-min one-month follow-up survey (completed online six weeks post-intervention) and a final 20-min follow-up survey two months later (completed online 14-weeks post-intervention). See Fig. 1 for RCT study flow. Participants receive a $20 gift card (multiple options to choose from such as Amazon, clothing stores, and coffee shops) for each of the three 20-min surveys. For each of the 28 two-minute daily surveys they complete, participants will receive $2 added to a gift card balance, for a total of $56 if all daily surveys are completed. It is made clear to participants that incentives are provided for completing the surveys, not for completing the intervention (or the control condition). All procedures have been approved by the Institutional Review Board at the university where the research is being conducted.

### Development of the intervention

#### Overview

The intervention, called Pregaming Awareness in College Environments (PACE), is comprised of a theoretically-informed, brief, accessible, and personalized intervention to address pregaming drinking among college students that is based on empirically-supported intervention components. It is tailored toward an individual’s personal goals, beliefs (perceptions, expectancies, self-efficacy), and behavior (protective strategies), focusing on the core components of brief interventions that mediate the effects of multiple component intervention programs (e.g., correcting perceived norms, use of protective behavioral strategies, increased self-efficacy, challenging expectancies, BAL feedback) [17, 18]. Content was informed by the BASICS approach [61], which is based on aspects of both Motivational Interviewing [62] and relapse prevention [63] and rooted in a harm reduction framework [15, 16]. BASICS for general drinking has been efficacious when delivered in individual and group formats [64, 65] and recently has been adapted for use on mobile phones [66]. Researchers found in a study of 94 heavy drinking college students that components of the BASICS intervention in a mobile format led to limited drinking during the 14 days of the study [66]. Yet, the intervention failed to demonstrate one-month effects on heavy drinking behavior compared to control. It is possible that minimal lasting effects were found after the intervention ended due to lengthy, non-targeted content (e.g., participants received a mean of 23 modules, 3–5 min each, with additional focus on smoking cessation). The innovation in the PACE intervention comes from its targeting of pregaming behavior directly, with components of BASICS modified to address this risky drinking practice. PACE presents videos with a female narrator, combined with interactive activities to engage participants and help them consider making changes to their pregaming behavior.

#### Format of the intervention

Nearly all college students own and use smartphones regularly [67]. Young adults report checking these phones constantly throughout the day at an average of 74 times per day [68]. Moreover, alcohol interventions based on BASICS components that are delivered online through computers, tablets, or mobile phones have shown efficacy and are a means to reach individuals with intervention efforts that may not have otherwise sought in-person care [69, 70]. Thus, we opted to deliver PACE to participants on mobile phones and created a mobile-friendly website to host the program. Students could log in and view the program, which takes an average of 25 min to complete. The mobile format increases access to the intervention, without need for in-person facilitator delivery or use of a desktop or laptop computer.

#### Intervention content

PACE begins with definitions and activities to help students better understand standard drinks (e.g., 12 oz of beer or one shot of most liquors), BAL, and alcohol metabolism rates. Students learn that alcohol takes time to be processed; thus, when consuming many drinks in a short period of time, this can place them at higher than anticipated levels of intoxication once arriving at the event. Often students do not feel the full effects of pregaming until they arrive at their destination [39, 44]. Participants receive BAL feedback and learn about alcohol’s biphasic curve and the point of diminishing returns (BAL of 0.05–0.06), where the “good things” about alcohol (e.g., feel relaxed and social) are maximized and there is *less* (not no) likelihood of experiencing the “not so good things” (e.g., consequences). This is important as students reach high BALs even before leaving for the night out; as BALs rise, potential for consequences increases [35, 49].

Students then learn that students at their school pregame less frequently and drink significantly less during pregaming than they perceived. Research shows that students have misperceptions of pregaming perceived norms and such misperceptions associate with their own pregaming drinking behavior [71]. Prior work has shown that reductions in perceived norms are one of the driving components of change in brief interventions with students, [18] and that reductions in tailgating norms (i.e., tailgating being one of the many contexts where pregaming occurs), specifically, mediated changes observed in a tailgating-focused brief intervention with students during a football game on campus [60]. Thus, during PACE, students are asked about the typical pregaming behavior (frequency in past 30 days, amount consumed per pregaming occasion) of their peers on campus, as well as how much they drink themselves. Graphs with descriptions, narration, and the source of the norms are presented on screen, with content showing the discrepancies between one’s own perceptions and actual norms, as well as between one’s own use and actual norms. Students see pregaming norms for both males and females on campus. This is followed by a video describing the theory behind how social norms work to perpetuate heavy drinking in college [72]. Campus norms were collected in the spring of 2019 in a first phase of the study, among 527 students from the university recruited through a random list from the university registrar. This sample was similar in demographics to the larger university community (mean age 20; 62% female, 56% racial/ethnic minority students), with 69% reporting pregaming behavior. Details about the Phase 1 norms documentation can be found elsewhere [73].

PACE content also focused on goals for the night (i.e., reasons for pregaming) and students learn that they can get what they want out of the night (e.g., feel relaxed, be more social) by drinking moderately or not at all during pregaming. Relatedly, participant’s pregaming-specific expectancies and beliefs that pregaming will make their night better are challenged through a presentation on alcohol placebo studies, where students hear about experiments where college students display the social effects of drinking even without consuming actual alcohol. This is important because students with positive outcome expectancies (e.g., it would be easier to talk to people) are more likely to pregame, and pregaming mediates the relationship between expectancies and hazardous drinking [74]. Participant’s drink refusal skills are reviewed with alternate strategies to use if feeling pressured to drink heavily during pregaming, as greater drink refusal self-efficacy associates with less pregaming [75]. As in a relapse prevention approach, which targets the people, situations, and feelings that may lead one to drink heavily [63], risky situations specific to the students (e.g., when in a large group, when getting ready to meet a potential romantic partner later that night) are reviewed. Protective drinking strategies specific to pregaming [73] are selected by the students and they are asked to try during their next pregaming event strategies they do not normally use.

The intervention concludes with a video summarizing the content and a personalized feedback sheet with resources, that also gets emailed to participants. The personalized feedback sheet contains the information from the intervention in a format viewable at a later time, as well as resources for seeking help on campus and in the community for drinking, sexual violence, and mental health.

### Beta test of the intervention

Participants completing the norms documentation survey were asked at the end of the survey if they would be interested in attending a focus group to offer feedback on the intervention once it was completed. Of the 527 participants, 75 met eligibility criteria and expressed interest in attending a focus group. In August 2020, we invited these 75 participants and obtained consent from 13 of them to review the first draft of the PACE intervention and provide feedback. These participants attended one of three online focus groups to provide feedback to our research team on what they liked and did not like, what could be improved regarding content and functionality, and ideas for improving engagement with the program. Focus groups were conducted online rather than in person as initially proposed, due to COVID-19 pandemic safety protocols. Focus group participants were provided with a $50 Amazon gift card.

Focus group participants’ feedback was primarily positive and generally focused on what they liked about the PACE intervention. Still, we prodded students to generate suggestions for improvement. Feedback that could feasibly be addressed within the scope of the budget was incorporated in the intervention and prepared for a final version to test in the RCT. Suggestions included modifying the graphs displaying the normative drinking patterns to improve readability, adding brief text introductions to each section to facilitate fluidity between sections, modifying images used for standard drinks (including adding an image of sake), adding additional protective strategies (e.g., avoiding use of motorized scooters after pregaming), correcting a few typos and modifying some color schemes, and adding additional campus-specific resources to the resources page.

### Control condition

Participants in the control condition of the RCT will be asked to view a series of text-based slides regarding general drinking behavior. These slides were accessible to be viewed on mobile phones, with content based on information obtained from the Rethinking Drinking website from the National Institute on Alcohol Abuse and Alcoholism (NIAAA).

### Analytic plan

#### Main effects

Main effects of the intervention will be evaluated for pregaming drinking, general drinking (i.e., drinks consumed both during and after pregaming), and alcohol-related consequences in the immediate term and short-term. In the immediate term, we will evaluate whether intervention participants pregame less frequently (i.e., fewer days per week), reach lower BALs on pregaming days, and consume fewer drinks during pregaming from the two weeks prior to the intervention to the two weeks post-intervention than those in the control condition. Estimated BALs will be calculated using Widmark’s formula, which is the standard method for estimating BAL (using sex, weight, amount consumed, time). We will also evaluate whether, compared to control, intervention participants drink fewer days overall (i.e., pregaming days and non-pregaming days), consume fewer drinks over the course of each drinking day, and report fewer consequences on drinking days from the two weeks prior to the intervention to the two weeks post-intervention. In the short term (baseline to six and 14-weeks post-intervention), we will evaluate main effects of the intervention on pregaming frequency (i.e., pregaming days in the past 30 days) and pregaming quantity (i.e., typical amount consumed during pregaming on pregaming days in the past 30 days). We will also evaluate main effects of the intervention on overall drinking days (i.e., pregaming days and non-pregaming days) in the past 30 days, average consumed on a typical drinking day in the past 30 days, and number of alcohol-related consequences experienced in the past 30 days.

#### Moderation

We will evaluate moderation by augmenting main effect models with interactions between four moderators of interest and the intervention. Significant interactions with sex, for example, will be indicative of an effect modification where the impact of the intervention can be different for men and women even if both groups realize a significant impact of the intervention. We will test four moderators of intervention efficacy: sex, age, baseline hazardous drinking scores on the Alcohol Use Disorders Identification Test (AUDIT) [76], and baseline motivation to change drinking. These moderators were selected based on the pregaming literature and to help determine the feasibility of this approach in spite of variations in behavior during the event. First, female students have been found to be at particular risk from pregaming, including higher pregaming BALs and subsequent hospitalizations [40, 49, 52, 77–79]. Thus, we hypothesize that women will benefit most from the intervention. Second, though there are few differences observed in pregaming frequency between students under 21 and 21 or older, students under age 21 have reported reaching higher BALs during pregaming than of-age students and are hypothesized to benefit most [36, 80]. Third, baseline levels of hazardous drinking will also be explored as a moderator, as heavier global drinkers drink more during pregaming [41, 81–83]. We hypothesize baseline heavier drinkers will benefit most.

#### Outcome measures

On all surveys, we will define pregaming behavior for participants as the following: “When we ask you about pregaming (a.k.a., prepartying), we are talking about the consumption of alcohol prior to attending an event or activity. For example, drinking before going to a party, bar, concert, sporting event, date, meeting, or any other event or activity at which more alcohol may or may not be consumed. This can be an event that has a large number of people or very few people.” Participants will also be provided with a graphic depicting standard drinks (i.e., 12 oz of beer with 5% alcohol/volume, 8–9 oz of craft beer with approximately 7% alcohol/volume, 4–5 oz of wine with approximately 13% alcohol/volume, 12 oz of hard seltzer with 5% alcohol/volume, 1.5 oz of 80 proof liquor with 40% alcohol/volume in either a shot glass or in a mixed drink).

#### Baseline and follow-up surveys

On the screening questionnaire, participants will be asked, “During the past 30 days, how often did you engage in pregaming,” with response options of never, just once, a couple of times, about once per week, a couple of times per week, and daily or almost daily. Those endorsing about once per week or more will screen into the study and complete the baseline survey. Overall drinking frequency will be assessed with an item asking, “During the past 30 days, how many days did you have at least one drink of any alcoholic beverage, such as beer, wine, hard seltzer, mixed drinks, or shots of liquor,” with response options from 0 to 30 days. To assess overall drinking quantity, participants will then be asked to consider their typical drinking behavior over the past 30 days with, “During the past 30 days, on the days when you drank, about how many drinks did you drink on average,” with response options from 0 to 30 drinks. Pregaming frequency will then be asked with the item, “During the past 30 days, how many days did you engage in pregaming,” with response options from 0 to 30 days. Pregaming quantity will be assessed with an item asking, “During the past 30 days, on the days when you drank, about how many drinks did you drink during pregaming,” with response options from 0 to 30 drinks. Alcohol consequences will be assessed with the Brief Young Adult Alcohol Consequences Questionnaire (B-YAACQ) [84, 85], where participants will indicate which (yes/no) of 24 alcohol-related consequences have happened to them in the past 30 days (e.g., I drove a car when I knew I had too much to drink to drive safely, I did not remember large stretches of time while drinking heavily). Participants will indicate race/ethnicity and class year (for descriptive purposes), age and biological sex (for moderation analyses), weight (for calculating estimated BAL), complete the 10-item AUDIT [76] (for moderation analyses), and indicate how motivated they are to drink less using a change ruler (scale from 0–10) modified from other work [86, 87] (for moderation analyses). Follow-up surveys will also ask intervention and control participants how long they spent viewing the content and whether they returned to review the content after initial viewing. Back-end data connected to PIN codes can also be used to determine whether participants finished viewing the intervention or control content or only completed a portion of either.

#### Daily surveys

Daily surveys will be delivered in the morning and ask about the day before. On the daily surveys, participants will first be asked if they drank yesterday (yes/no). If so, they will be asked how much they drank overall with an item assessing, “How may drinks did you have total yesterday,” with response options of 0 to 30 drinks. They will then be asked if they pregamed yesterday; if so, they will be asked, “How many of the [drinks they had overall] did you have while pregaming, with response options from 0 and capping at the overall amount they indicated for that day. On days they drank (i.e., pregaming or non-pregaming day), they will then be asked if they experienced (yes/no) any of the 24 BYAACQ consequences that day. We will evaluate any of the 24 consequences as an outcome (summed score ranging from 0 to 24).

## Discussion

This brief, personalized, and easily accessible mobile phone-based intervention focused on pregaming is proposed to help college students develop and use drinking-reduction skills to limit the amount they drink while pregaming. The advent of smartphones has led to increased intervention opportunities to target risky behaviors among those who may not otherwise have sought help for their drinking [88, 89]. As college students typically do not pursue treatment to address alcohol use despite engaging in frequent heavy drinking [90, 91], having a brief intervention available to them that is both easy to use and engaging is essential. Smartphone and app-based interventions have gained popularity, with the few available ones demonstrating promise on reducing alcohol use outcomes [66, 92]. Similar smartphone-based text message interventions have also shown promise of efficacy with college drinkers [93]. Though hundreds of alcohol apps exist in the public domain for download onto smartphones, few if any include empirically-supported behavioral change techniques or have demonstrated efficacy at actually reducing drinking [94, 95]. For example, apps with BAL information are available for download, but they provide inaccurate estimates, misleading information (e.g., asking users to blow into the phone’s microphone to estimate BAL), do not provide personalized feedback, and are not empirically based [96–98]. Importantly, the intervention we designed and propose to test in the RCT represents one of the first to address pregaming specifically. That is, for this project, the intervention is specifically designed to focus upon the drinking behavior that is known to be perhaps the riskiest drinking practice for many students, and content is personalized to help students address their own personal risk factors for drinking during pregaming. The content may help address underlying traits associated with problem drinking in general (e.g., practicing refusal skills for those with little refusal self-efficacy) and prepare students who pregame less frequently to avoid problems that may emerge on an impromptu pregame night involving greater consumption than what is typical. Given that upwards of 80% of student drinkers report pregaming behavior in the past month alone [39], the intervention has broad applicability to the majority of college students for both intervention and prevention efforts.

The proposed research is innovative in four main respects. First, there has been a call for research and interventions targeting high intensity drinking [51] and, as stated, this is among the first intervention studies to directly target pregaming–a popular and risky aspect of the college drinking culture that cuts across specific contexts leading to high intensity drinking and resulting problems. Second, by using a pregaming-specific mobile-based intervention, the intervention expands on promising preexisting smartphone app-based brief interventions that target non-specific events, global in-person and web-based approaches with small effect sizes, and event-specific prevention programs that target a single risky event (e.g., tailgating). Third, the smartphone-based intervention can be widely available to students outside of research settings to increase access to a theoretically-informed and evidence-based brief intervention. If found to be efficacious, it has the potential to reach millions of college students, perhaps as a clinical tool used by college counseling centers, an intervention for adjudicated students on campus, or modified for use as a brief orientation program for incoming first year students to prevent pregaming during the high-risk initial weeks on campus. The easy-to-use tool could be adopted for use beyond college students with high school students and non-college young adults who also report frequent pregaming [41, 47, 83]. Fourth, the pregaming intervention is tailored toward the individual student, in that it targets personalized beliefs and behaviors known in the literature on brief college drinking interventions to lead to positive outcomes, such as by targeting one’s positive expectations to result from pregaming, correcting misperceived norms of pregaming, and encouraging use of protective strategies during prepartying [17, 18].

## Limitations and alternative methods considerations

We have considered potential limitations of the research design and planned for them where possible. First, by design, participants in both intervention and control groups complete daily assessments of their drinking behaviors for 28 days. Regarding assessment reactivity during the 28 days of these daily surveys, research has cited minimal reactivity to daily diary assessments; there is no evidence that prompting individuals to assess their alcohol use leads one to drink [66, 99]. However, repeatedly self-reporting on drinking (or self-monitoring as it has been called) can be a form of intervention that could lead to reductions in drinking [100]. Though our analytic plan calls for analyses of pre-intervention and post-intervention daily data, it is possible the control group may be impacted to change their drinking by this self-monitoring. Thus, any significant intervention effects we find will need to be interpreted as occurring over the effects of simply self-monitoring.

Second, the intervention is quite brief (20–30 min) by design. It is delivered on one occasion to capitalize on the innovation and brevity of this approach. Other brief interventions are delivered over several drinking days, but these can be burdensome, and the feasibility of such an approach is low. Therefore, we desire to show support for a one-time event-specific approach–the effects of which are anticipated to generalize to future pregaming events once individuals learn to moderate their pregaming drinking effectively. As this is the first randomized controlled trial test of the intervention, we want to determine if the intervention targeting pregaming alone is efficacious. If it is not, or if it is only efficacious for certain students, then future work can refine this initial approach to enhance the intervention to possibly include repeated delivery after pregaming or perhaps during multiple pregaming events.

Lastly, though the PACE intervention was completed and ready to be implemented in the RCT in late 2019 (with a plan to enroll participants starting in the spring semester of 2020), the COVID-19 pandemic and stay-at-home orders prevented us from starting the study on time. We waited until students were back on campus and living in residence halls again; thus, the study began with recruitment in the fall semester of 2021.

## Conclusion

In conclusion, this pregaming-specific intervention has potential for impacting heavy college drinking as it targets a popular dangerous activity that, if reduced, could possibly lead to reduced drinking overall. This study will inform future grant efforts and the smartphone-based app could be delivered to millions of pregaming college students, at any desired interval, for a host of qualifying reasons, to prevent heavy pregaming drinking for a fraction of the cost it would take to intervene individually with students who have established heavy drinking patterns.

## Abbreviations

AUDIT: Alcohol Use Disorders Identification Test
BAL: Blood alcohol level
BASICS: Brief Alcohol Screening and Intervention for College Students
B-YAACQ: Brief Young Adult Alcohol Consequences Questionnaire
MI: Motivational Interviewing
NIAAA: National Institute on Alcohol Abuse and Alcoholism
PACE: Pregaming Awareness in College Environments
RCT: Randomized controlled trial
